# Carbapenem-resistant *Acinetobacter baumannii* from Brazil: role of *car*O alleles expression and *bla*_OXA-23_ gene

**DOI:** 10.1186/1471-2180-13-245

**Published:** 2013-11-06

**Authors:** Erica Lourenço Fonseca, Erica Scheidegger, Fernanda S Freitas, Rosângela Cipriano, Ana Carolina P Vicente

**Affiliations:** 1 Laboratório de Genética Molecular de Microrganismos, Instituto Oswaldo Cruz, FIOCRUZ, Rio de Janeiro, Brazil; 2 Departamento de Medicina 1, Universidade Federal do Maranhão, São Luís, Maranhão, Brazil

**Keywords:** *Car*O, OXA-23, Expression, Imipenem resistance, Acinetobacter, Brazil

## Abstract

**Background:**

Carbapenems are the antibiotics of choice to treat infections caused by *Acinetobacter baumannii*, and resistance to this class can be determined by loss of membrane permeability and enzymatic mechanisms. Here, we analyzed the basis of carbapenem resistance in clinical *A. baumannii* isolates from different Brazilian regions.

**Results:**

The analyses addressed the carbapenemase activity of OXA-23, CarO expression and alterations in its primary structure. Susceptibility test revealed that the strains presented the COS (Colistin-Only-Sensitive) profile. PCR and sequencing showed the presence of the chromosomally-encoded *bla*_OXA-51_ in all isolates. The majority of strains (53%) carried the carbapenemase *bla*_OXA-23_ gene associated with IS*Aba1*. The Hodge test indicated that these strains are carbapenemase producers. PFGE revealed 14 genotypes among strains from Rio de Janeiro and Maranhão. The influence of *car*O on imipenem resistance was evaluated considering two aspects: the composition of the primary amino acid sequence; and the expression level of this porin. Sequencing and *in silico* analyses showed the occurrence of CarOa, CarOb and undefined CarO types, and Real Time RT-PCR revealed basal and reduced *car*O transcription levels among isolates.

**Conclusions:**

We concluded that, in general, for these Brazilian isolates, the major carbapenem resistance mechanism was due to OXA-23 carbapenemase activity and that loss of CarO porin plays a minor role in this phenotype. However, it was possible to associate the *car*O alleles and their expression with imipenem resistance. Therefore, these findings underline the complexity in addressing the role of different mechanisms in carbapenem resistance and highlight the possible influence of CarO type in this phenotype.

## Background

*Acinetobacter baumannii* has been considered one of the major nosocomial pathogens worldwide, and it is included in the group of ESKAPE bugs, in which some lineages effectively escape the action of antibacterial drugs [1]. Carbapenems remain the antibiotic of choice to treat *A. baumannii* and other Gram-negative infections due to both a wider spectrum of antibacterial activity and less frequent side effects. However, their overuse and misuse have selected for nosocomial isolates presenting intrinsic and acquired multidrug resistance determinants [2]. It has been considered that resistance against carbapenems is, in itself, sufficient to define an *A. baumannii* as highly resistant [3]. The molecular basis of carbapenem resistance in this species has been ascribed to the recruitment and production of carbapenem-hydrolysing class D β-lactamases (CHDLs) and, to a lesser extent, of metallo-β-lactamases (MBLs). In *A. baumannii*, the CHDLs can be intrinsic (OXA-51-like) or acquired (OXA-23-like, OXA-24-like and OXA-58-like) [3]. Although these enzymes weakly hydrolyze carbapenems, they can confer high resistance when *bla*_OXA_ genes are overexpressed, as a result of their association with mobile elements, such as IS*Aba1*, which carries a strong promoter [4]. The *bla*_OXA-23_ gene, in association with IS*Aba1*, is spread in *A. baumannii* worldwide, and it is the most prevalent OXA allele in isolates from Brazil [3,5-7].

In addition to this enzymatic resistance, the loss of membrane permeability, due to alterations in specific porins, is an intrinsic carbapenem resistance mechanism in *A. baumannii*. The 25/29 kDa heat-modifiable carbapenem-associated outer membrane protein (CarO) is a porin with a β-barrel topology. This channel allows the selective uptake of amino acids and imipenem (but not meropenem) due to its structural conformation and to the presence of an imipenem binding site [8,9]. This site was not found in any other OMP, and thus, CarO protein is considered a preferred uptake channel for this antibiotic. Based on variable domains of CarO, this channel is classified in two groups, CarOa and CarOb, with high affinity to imipenem. Moreover, CarOb was showed to be twice more specific for imipenem than the CarOa. In this way, *car*O gene alterations, as disruption by insertion sequences, changes in the primary structure - mainly in the imipenem binding site - or decreased expression, would have a dramatic impact on the entry of imipenem in the cell, thus contributing to resistance to this antibiotic [9]. Concerning the participation of CarO porin in the resistance phenotype, few descriptive studies have only shown the presence/absence of the gene and protein, resulting in a lack of studies showing the association of the *car*O alleles and their expression with the resistance phenotype.

Brazil is a continental country and, concerning resistance in *A. baumannii*, the studies have been concentrated in the Southeast region [5,7], which could not be representative of the *A. baumannii* diversity as showed previously [10]. Therefore, the role of CarOa and CarOb in the differential imipenem uptake, and the current diversity of *car*O alleles available in the GenBank lead us to investigate these aspects in a set of carbapenem-resistant clinical *A. baumannii* strains from two Brazilian states placed ~3,000 Km apart and with distinct ecological and social contexts. Moreover, the prevalence and participation of carbapenemases in this phenotype was also evaluated.

## Results and discussion

All the 28 strains were resistant to, at least, meropenem. Most of the strains from both Rio de Janeiro and Maranhão were resistant to ceftazidime, cefepime, ciprofloxacin, trimethoprim, amikacin and streptomycin (Table 1). A considerable discrepancy was observed between strains from Rio de Janeiro and Maranhão concerning the resistance to aztreonam, gentamicin, piperacillin/tazobactam, ertapenem, and imipenem (Table 1). Three/thirteen (23%) strains from Rio de Janeiro were resistant to imipenem contrasting with 13/15 (86.6%) resistant strains from Maranhão. All strains from this study were susceptible to tigecycline and colistin (MICs ranging from 0.25 to 0.50 μg/mL), and strains resistant to imipenem presented MICs ≥ 32 μg/mL.

**Table 1:** **Genetic and phenotypic features of** ***A. baumannii*** **Brazilian strains analyzed in this study**

**Strains (n)**	**PFGE**	**RQ of *car*** **O**	**CarO classification**	***bla***^**a**^_**OXA**_	**IS** ***Aba1***	**IS** ***Aba1*** **+** ***bla***_**OXA**_^**b**^	**Hodge test**	**Resistance phenotype**	**MIC of Imipenem (μg/mL)**
AC2-RJ (1)	B	0.41	CarOa	*bla*_OXA-23_	+	+	+	ATM CAZ CIP MEM IPM EPM TMP GEN AMK STR	≥ 32
AC1-RJ (1)	A	1.33	CarOa	*bla*_OXA-13_	+	-	-	ATM CAZ FEP CIP MEM TMP STR	1.0
AC3-RJ (3)	C	1.21	CarOa	*bla*_OXA-66_	+	-	-	ATM CAZ FEP CIP MEM TMP AMK STR	1.0
AC10-RJ (1)	F	1.23	CarOa	*bla*_OXA-10_	+	-	-	ATM CIP MEM TMP GEN AMK STR	1.0
AC6-RJ (3)	D	1.07	Undefined CarO	*bla*_OXA-13_	+	-	-	ATM CAZ FEP CIP MEM IPM EPM TZP TMP GEN AMK STR	32
AC9-RJ (1)	E	0.10	CarOb	*bla*_OXA-10_	+	-	-	ATM CIP MEM TMP GEN STR	0.25
AC16-RJ (2)	G	0.86	CarOb	*bla*_OXA-10_	+	-	-	ATM CAZ FEP CIP MEM TZP TMP AMK STR	1.5
AC72-RJ (1)	H	0.94	CarOa	*bla*_OXA-23_	+	+	+	ATM CAZ CIP MEM IPM EPM TMP AMK STR	≥ 32
AC60-MA (1)	L	0.05	Disrupted by *IS*Aba1	-	+	-	-	MEM EPM	0.20
AC62-MA (2)	J	0.45	CarOa	*bla*_OXA-23_	+	+	+	ATM CAZ FEP CIP MEM IPM EPM TZP TMP AMK STR	≥ 32
AC65-MA (1)	M	0.50	CarOb	*bla*_OXA-23_	+	+	+	ATM CAZ FEP CIP MEM IPM EPM TZP TMP GEN AMK STR	≥ 32
AC67-MA (8)	I	0.65	Undefined CarO	*bla*_OXA-23_	+	+	+	CAZ FEP CIP MEM IPM EPM TZP TMP GEN AMK STR	≥ 32
AC70-MA (1)	N	1.55	CarOa	*bla*_OXA-66_	+	-	-	ATM CAZ FEP CIP MEM EPM TZP TMP GEN AMK STR	1.0
AC71-MA (2)	K	0.45	Undefined CarO	*bla*_OXA-23_	+	+	+	CAZ FEP CIP MEM IPM EPM TZP TMP GEN AMK STR	≥ 32
ATCC 19606		1.00	CarOa						0.25

^a^all isolates also harboured the *bla*_OXA-51_ gene.

^b^no *bla*_OXA-51_ was associated with IS*Aba1*.

*ATM* aztreonam, *CAZ* ceftazidime, *FEP* cefepime, *CIP* ciprofloxacin, *MEM* meropenem, *IPM* imipenem, *EPM* ertapenem, *TZP* piperacillin/tazobactam, *TMP* trimethoprim, *GEN* gentamicin, *AMK* amikacin, *STR* streptomycin.

All strains harboured the chromosomally-encoded *bla*_OXA-51_ gene, however, the IS*Aba1* was not associated to this allele in any strain. In fact, the IS*Aba1*-*bla*_OXA-51_ combination is not frequent [5], although a previous study had found this association in the Southern region of Brazil [7]. Additionally to *bla*_OXA-51_, 13/15 (86.6%) strains from Maranhão carried also the CHDL *bla*_OXA-23_ gene, while only 2/13 (15%) strains from Rio de Janeiro (AC2-RJ and AC72-RJ) harboured this allele. Spite the low prevalence of *bla*_OXA-23_ in AC-RJ strains (this work) and in São Paulo [6], it is the most prevailing and disseminated allele in Brazil. All *bla*_OXA-23_ from both Maranhão and Rio de Janeiro were preceded by IS*Aba1* as found in previous works from several Brazilian states [5,7]. Therefore, the high imipenem resistance (MIC, ≥ 32 μg/mL) presented in all strains harbouring *bla*_OXA-23_ can be due to its overexpression driven by the IS*Aba1* promoter [4]. All strains from Rio de Janeiro, except by AC2-RJ and AC72-RJ, carried *bla*_OXA_ alleles of narrow resistance spectrum (*bla*_OXA-13_, _-10_ or _-66_), none of them associated with IS*Aba1,* spite its presence in AC-RJ strains (Table 1). The isolates were negative for MBL genes, *bla*_OXA-58-like_ and *bla*_OXA-24-like_ alleles. In summary, our results revealed that only OXA with carbapenemase activity (*bla*_OXA-23_) was associated with IS*Aba1* in the strains analyzed here, and that all of them were resistant to carbapenems. These results are summarized in Table 1.

The Hodge test was in agreement with the carbapenemase activity and the consequent carbapenem resistance, where positive results were obtained only for the strains carrying IS*Aba1*-*bla*_OXA-23_, while negative results were observed for strains harbouring OXA alleles with no or a weak carbapenemase activity. Thus, these results reflect the OXA-23 overexpression driven by the strong promoter [4,11] found in IS*Aba1* (Table 1).

*A. baumannii* has a clonal structure population [12], however, several unrelated carbapenem-resistant lineages have been reported in Brazil [13,14]. In the present work, macrorestriction analyses revealed eight genotypes in Rio de Janeiro (A-H) and six genotypes in Maranhão (I-N), totalizing 14 clones, none of them related to the international clones. In Maranhão, the majority of strains (8/15) belonged to genotype I, however in Rio de Janeiro there was no predominant genotype. In this study, the *bla*_OXA-23_ gene was distributed in several genotypes, while in São Paulo this allele occurred in only one genotype [6]. These findings show the diversity of clinical *A. baumannii* circulating in Brazil. One strain of each genotype was selected as a representant to perform further analyses of *car*O transcription and gene sequencing (Table 1).

Considering CarO, imipenem resistance can be influenced in three aspects: i) the occurrence of gene disruption resulting in the lack of porin formation in the outer membrane; ii) amino acid changes in both CarOa and CarOb that could account for an altered porin with low or no affinity for the antibiotic and/or; iii) decrease expression of the *car*O gene that causes a low density of channels. Therefore, to fully investigate these aspects, it is necessary to assess the *car*O nucleotide and deduced amino acid sequences and its expression. However, there is a lack of this set of data when the role of *car*O in imipenem resistance is addressed.

Here, in order to raise all these information concerning *car*O, we analyzed the expression of this gene by relative quantification, and the nucleotide/predicted amino acid sequences were determined to type the CarO (CarOa or CarOb) and to evaluate gene disruption by IS. The *car*O sequences from AC-RJ and AC-MA presented substitutions without the presence of frameshifts and/or premature stop codons when compared to CarO of *A. baumannii* ATCC 19606. Analyses of the *car*O promoter region showed that all strains harbour the canonical sequence as described previously [15].

**Figure 1:**
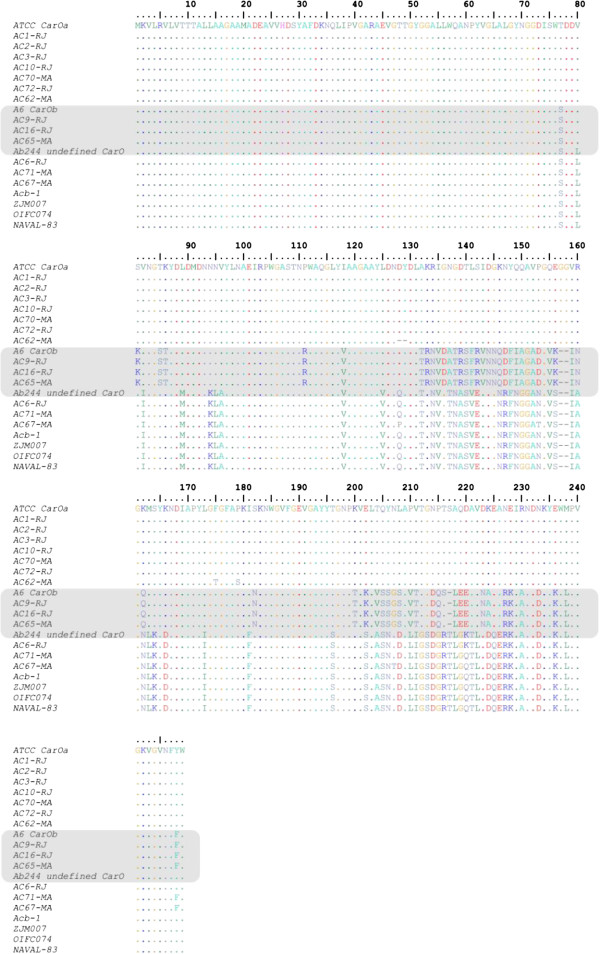
**Alignment of *A. baumannii *CarO amino acid sequences from this study and available in GenBank.** The ATCC 19606 [GenBank: ZP_05828783.1] and A6 [GenBank: ADQ27797.1] CarO sequences were selected as the representants of the CarOa and CarOb groups, respectively. Ab244 [GenBank: AAV80243; Argentina], Acb-1 [GenBank: ABG27024; Leban], ZJM007 [GenBank: AFH56947; China], OIFC074 [GenBank: EKL39362; Germany] and Naval-83 [GenBank: EKL48836; United States] were representants of different undefined CarO isoforms. The numbers correspond to amino acid positions. The gray box labels the CarOb group.

The predicted amino acid sequence showed that CarOa and CarOb were distributed among isolates from Maranhão and Rio de Janeiro. Moreover, blastX analyses revealed CarO sequences with amino acid differences considering both CarOa and CarOb, which was named here undefined CarO (Table 1 and Figure 1). The undefined CarO porins (AC6-RJ, AC-67-MA and AC71-MA) presented one to five amino acid differences among each other, with 70% and 74% identity with CarOa and CarOb, respectively. One of these, from AC6-RJ, is identical to a CarO previously identified in Argentina [GenBank: AAV80243] [15]. AC67-MA and AC71-MA carried two new undefined CarO with 98% and 99% amino acid identity, respectively, with CarO sequences identified in strains from Germany, United States, Leban and China [GenBank: EKL39362, EKL48836, ABG27024, AFH56947, respectively] (Figure 1). Therefore, taking into account the observed CarO diversity, and that the CarO primary structure influence the imipenem affinity and, consequently the imipenem resistance, the impact of other isoforms on this phenotype has to be investigated.

Considering the imipenem-susceptible strains, relative quantification assay revealed that AC1-RJ (CarOa), AC3-RJ (CarOa), AC10-RJ (CarOa), AC16-RJ (CarOb), and AC70-MA (CarOa) had a basal transcription level of *car*O as ATCC 19606 (Table 1). For this group of strains, the *car*O transcript amount and the type of CarO were in agreement with the imipenem susceptibility phenotype (Table 1). Moreover, these strains did not produce carbapenemases. However, the *car*O expression did not correlate with this phenotype in the imipenem-susceptible AC60-MA and AC9-RJ strains. In AC60-MA, which is highly susceptible to imipenem (MIC, 0.20 μg/mL), the *car*O gene was disrupted by the IS*Aba1* (Table 1). Therefore, the porin loss in this strain was not evoking resistance, indicating the participation of other mechanisms in the imipenem uptake. Similarly, AC9-RJ, susceptible to imipenem (MIC, 0.25 μg/mL), presented a 10-fold decrease in *car*O expression. It can be suggested that, apparently, this 10-fold expression reduction was not enough to cause the total loss of porin formation. Interestingly, AC9-RJ has a porin from the CarOb group, which is more specific towards imipenem than CarOa [9]. Thus, even in lower amounts of the porin, the imipenem uptake can be occurring due to CarO structural conformation in this strain, explaining its susceptibility to imipenem.

Considering the imipenem-resistant strains, AC2-RJ (CarOa), AC62-MA (CarOa), AC65-MA (CarOb), AC67-MA (undefined CarO) and AC71-MA (undefined CarO) had low *car*O transcript amounts (Table 1). The exception was AC72-RJ, which presented basal *car*O transcription. All of them produce the CHDL OXA-23, and therefore, it was not possible to establish the contribution of CarO in this resistance phenotype (Table 1). Conversely, the imipenem-resistant AC6-RJ strain (MIC, 32 μg/mL), presented basal *car*O transcription level (1.07-fold), and no carbapenemase production was detected (Table 1). As showed above, the AC6-RJ harbours an undefined CarO type, presenting 70% and 74% amino acid identity with CarOa and CarOb, respectively. Therefore, these CarO could have an altered imipinem binding site, influencing the antibiotic affinity, resulting in the observed resistance. In fact, this undefined CarO from AC6-RJ was identical to that from Ab244 strain from Argentina, which has been shown to play an essential role in the selective uptake of L-ornithine, but not of imipenem [8,9].

Therefore, to correlate CarO with imipenem resistance, not only the expression has to be considered, but also the CarO primary sequence. The results obtained for AC6-RJ and AC9-RJ provide evidences of a possible correlation between CarO isoforms and imipenem resistance/susceptibility phenotype (Table 1). Moreover, PROVEAN analyses revealed that the amino acid substitutions (approximately 67 residues) found in the different undefined CarO identified in this study had a neutral impact in protein function. These results indicate that even with this high proportion of substitutions they are not interfering with CarO function and formation.

The particular role of some CarO (CarOa and CarOb) in the imipenem affinity had been experimentally determined by Catel-Ferreira and colleagues [9]. Vashist et al. [16] indirectly showed a correlation between expression, the CarO isoform and imipenem resistance based on proteomic approaches. Here, we directly determined the CarO isoforms by assessing the substitutions in the amino acid primary sequence and the transcription level of CarO. Also, Vashist and colleagues showed that altered CarO isoforms were upregulated while the native CarO (CarOa and CarOb) were downregulated in carbapenem-resistant *A. baumannii*. These results obtained at the post-translational level contrast with ours since no correlation between the CarO (native or undefined) and the transcription level (up and downregulation) was observed in this study (Table 1). This reflects that post-transcriptional events may be occurring during porin formation in the membrane, since in that work the authors analyzed the protein. Therefore, our study is the first to infer the role of CarO on imipenem resistance considering its expression and allelic diversity.

## Conclusions

Studies in distinct Brazilian geographic regions have already shown the prevalence of OXA-23 among the several CHDLs in imipenem resistant *A. baumannii* strains [5,7]. However, there is a lack of data concerning the influence of membrane permeability loss in this phenotype. We concluded that, spite of the carbapenemase production being the major carbapenem resistance mechanism in *A. baumannii* analyzed here, the amino acid composition of CarO porins can affects more drastically the imipenem resistance than the transcription level itself. Therefore, these findings highlight the possible role of CarO isoforms in this phenotype.

## Methods

### Bacterial strains and detection of carbapenem-resistance genes

The study included 28 clinical carbapenem-resistant *A. baumannii* belonging to the Bacterial Culture Collection of Environment and Health (CBAS) from the Oswaldo Cruz Institute (FIOCRUZ). The isolates were recovered from Maranhão in 2006 (n = 15) and Rio de Janeiro in 2008 (n = 13), and presented a COS phenotype, with the exception of AC60-MA strain. PCR and sequencing reactions were performed targeting the MBLs genes *bla*_IMP_ and *bla*_VIM_ alleles, *bla*_SPM-1_, and *bla*_NDM-1_; and the CHDLs *bla*_OXA-58-like_, *bla*_OXA-51_, *bla*_OXA-24-like_, *bla*_OXA-23_, *bla*_OXA-143_ and other OXA alleles (Table 2), alone and downstream IS*Aba1*. Also, the entire *car*O gene and its promoter region were evaluated. Primer list is detailed in Table 2.

**Table 2:** Primers used in this study

**Primers for PCR and sequencing**		
**Primer name**	**Primer sequence (5′ → 3′)**	**Target gene**
IMP F	GCATTGCTACCGCAGCAGAG	*bla*_IMP_ alleles
IMP R	GGTTTAAYAAAACAACCAC	
VIM F	TCGGAGAGGTCCGACTTTACC	*bla*_VIM_ alleles
VIM R	CCATTCAGCCAGATCGG	
SPM-1 F	ACGTTTTCGTCGTCACAG	*bla*_SPM-1_ gene
SPM-1 R	GTCCAGGTATAACAATTTTCG	
NDM-1 F	AATGGAATTGCCCAATATTATGC	*bla*_NDM-1_ gene
NDM-1 R	TCAGCGCAGCTTGTCGGC	
OXA-23 F	GATGTGTCATAGTATTCGTCG	*bla*_OXA-23_ gene
OXA-23 R	TCACAACAACTAAAAGCACTG	
OXA SET A F^a^	ATGAAAAAATTTATACTTCC	*bla*_OXA-24-like_ alleles
OXA SET A R	TTAAATGATTCCAAGATTTTC	
OXA SET B F	ACAGAARTATTTAAGTGGG	*bla*_OXA-51-like_ and *bla*_OXA-58-like_ alleles
OXA SET B R^a^	GGTCTACAKCCMWTCCCCA	
OXA SET C F	ATGAAAACATTTGCCGCATA	*bla*_OXA-10-like_ alleles
OXA SET C R	CGTTGTCTATATCCATGTTA	
ISABA1 F	GTGCTTTGCGCTCATCATGC	IS*Aba1*
ISABA1 R	CATGTAAACCAATGCTCACC	
ISABA1 F2^b^	AGTTGCACTTGGTCGAATGA	Detection of IS*Aba1* upstream *bla*_OXA-51_ and *bla*_OXA-23_
CARO F	GCTCACCTGATGCTGACATT	*car*O gene and its promoter
CARO R	TTGCTTCTTCAACAGCTTGG	
CARO F SQ	GCGGCTTACCTTGATAACG	*car*O gene sequencing
CARO R SQ	TTACGAGCTTCCGCATTTAC	
**Primers for relative quantification**		
TR CARO F	AGCTTTACTTGCTGCTGGTG	*A. baumannii car*O transcription
TR CARO R	CGAGCGCCTACTGGAATTA	
TR CPN60 F	TTGACCGTGGTTATATCTCTCC	*A. baumannii cpn*60 transcription
TR CPN60 R	CGGATTTTCAAGTTCAGCAG	

^a^ Used in combination to amplify the *bla*_OXA-143_ allele.

^b^used in combination with OXA SET B R and with OXA-23 R primers.

### Antimicrobial susceptibility testing

The resistance phenotype was obtained by the disc-diffusion method [17] for the following antibiotics: aztreonam, cefepime, ceftazidime, piperacillin-tazobactam, ciprofloxacin, trimethoprim, imipenem, meropenem, ertapenem, gentamicin, amikacin, streptomycin, colistin and tigecycline. The MIC was determined by E-Test method (Biomerieux) for imipenem and colistin.

### Evaluation of carbapenemase production

The carbapenem-resistant isolates were submitted to the modified Hodge test [18] in order to detect the carbapenemase production. In summary, the assay was carried out on Müeller-Hinton agar plates using ertapenem, meropenem and imipenem disks. The *bla*_SPM-1_ MBL-producing *Pseudomonas aeruginosa* (PS600) from CBAS and the *Escherichia coli* strain ATCC 25922 were used as positive and negative controls, respectively.

### PFGE genotyping

PFGE analyses were performed using *Apa*I (New England Biolabs) restriction enzyme in order to determine the genetic relationship among *A. baumannii* isolates. The genetic relatedness was attributed when the macrorestriction DNA patterns differed by fewer than three bands [19].

### Relative quantification of CarO porin

The *car*O transcription was evaluated by real time RT-PCR using Power-SYBR Green PCR Master Mix (Applied Biosystems). The single-copy housekeeping *cpn*60 gene from *A. baumannii*, coding for a 60-kDa chaperonin, was used as endogenous gene for normalization. The relative quantification (RQ) results were presented as ratios of normalized target gene transcription between the *A. baumannii* clinical isolates and the Type Strain ATCC 19606 (calibrator), which were obtained according to the following equation: RQ = 2^-∆∆CT^, where CT is the value corresponding to the crossing point of the amplification curve with the threshold; ∆CT = target CT or calibrator CT – endogenous CT; and ∆∆CT = target ∆CT – calibrator ∆CT. *car*O differential transcription of clinical strains relative to that of ATCC 19606 was considered significant when the ratios obtained between RQ values (RQ value of calibrator/RQ value of clinical strains) were ≥2.0, taking into account the standard deviation intervals.

### CarO amino acid sequence alignment and in silico analyses

The deduced amino acid sequences from the *car*O obtained in this study and from the CarOa (ATCC 19606 strain), CarOb (A6 strain) and several undefined CarO sequences available in GenBank were aligned using ClustalW. In order to verify whether the amino acid changes observed in the undefined CarO isoforms relative to the canonical CarOa are deleterious, *in silico* analyses was performed with the software PROVEAN (http://provean.jcvi.org/), which predicts if an amino acid substitution or indel has any impact on the biological function of a protein.

## Competing interests

The authors declare that they have no competing interests.

## Authors’ contributions

EL conceived and designed the study, carried out the experimental assays, analyzed and interpreted the data and drafted the manuscript. EMDS and FSF performed the experimental assays. RC provided the isolates and performed the preliminary microbiological tests. ACPV conceived, designed and supervised the study, and edited the manuscript. All authors read and approved the final manuscript.

## References

[B1] Boucher HW Talbot GH Bradley JS Edwards JE Jr Gilbert D Rice LB Scheld M Spellberg B Bartlett J Bad bugs, No drugs: No ESKAPE! an update from the infectious diseases society of America Clin Infect Dis 2009 48 1 12 10.1086/595011 19035777

[B2] Kuo HY Chang KC Kuo JW Yueha HW Liouf ML Imipenem: a potent inducer of multidrug resistance in *Acinetobacter baumannii* Int J Antimicrob Agents 2012 39 33 38 10.1016/j.ijantimicag.2011.08.016 21996406

[B3] Poirel L Nordmann P Carbapenem resistance in *Acinetobacter baumannii*: mechanisms and epidemiology Clin Microbiol Infect 2006 12 826 836 10.1111/j.1469-0691.2006.01456.x 16882287

[B4] Turton JF Ward ME Woodford N Kaufmann ME Pike R Livermore DM Pitt TL The role of IS*Aba1* in expression of OXA carbapenemase genes in *Acinetobacter baumannii* FEMS Microbiol Lett 2006 258 72 77 10.1111/j.1574-6968.2006.00195.x 16630258

[B5] Werneck JS Picão RC Girardello R Cayô R Marguti V Dalla-Costa L Gales AC Antonio CS Neves PR Medeiros M Mamizuka EM Elmor de Araújo MR Lincopan N Low prevalence of *bla*_OXA-143_ in private hospitals in Brazil Antimicrob Agents Chemother 2011 55 4494 4495 10.1128/AAC.00295-11 21849571 PMC3165347

[B6] Mostachio AK Levin AS Rizek C Rossi F Zerbini J Costa SF High prevalence of OXA-143 and alteration of outer membrane proteins in carbapenem-resistant *Acinetobacter spp*. isolates in Brazil Int J Antimicrob Agents 2012 39 396 401 10.1016/j.ijantimicag.2012.01.021 22455794

[B7] Pagano M Martins AF Machado AB Barin J Barth AL Carbapenem-susceptible *Acinetobacter baumannii* carrying the IS*Aba1* upstream *bla*_OXA-51-like_ gene in Porto Alegre, southern Brazil Epidemiol Infect 2012 19 1 4 10.1017/S095026881200074X PMC9152067 22717017

[B8] Mussi MA Relling VM Limansky AS Viale AM CarO, an *Acinetobacter baumannii* outer membrane protein involved in carbapenem resistance, is essential for L-ornithine uptake FEBS Lett 2007 581 5573 5578 10.1016/j.febslet.2007.10.063 17997983

[B9] Catel-Ferreira M Coadou G Molle V Mugnier P Nordmann P Siroy A Jouenne T Dé E Structure-function relationships of CarO, the carbapenem resistance-associated outer membrane protein of *Acinetobacter baumannii* J Antimicrob Chemother 2011 66 2053 2056 10.1093/jac/dkr267 21705362

[B10] Fonseca EL Fdos Freitas S Scheidegger ÉM Jacinto T Vicente AC Class 2 integrons in multidrug-resistant *Acinetobacter baumannii* circulating in different Brazilian geographic regions Int J Antimicrob Agents 2011 38 95 96 10.1016/j.ijantimicag.2011.03.013 21550785

[B11] Corvec S Poirel L Naas T Drugeon H Nordmann P Genetics and expression of the carbapenem-hydrolyzing oxacillinase gene *bla*_OXA-23_ in *Acinetobacter baumannii* Antimicrob Agents Chemother 2007 51 1530 1533 10.1128/AAC.01132-06 17220422 PMC1855470

[B12] Higgins PG Dammhayn C Hackel M Seifert H Global spread of carbapenem-resistant *Acinetobacter baumannii* J Antimicrob Chemother 2010 65 233 238 10.1093/jac/dkp428 19996144

[B13] Mugnier P Poirel L Naas T Nordmann P Worldwide dissemination of the *bla*_OXA-23_ carbapenemase gene of *Acinetobacter baumannii* Emerg Infect Dis 2010 16 35 40 20031040 10.3201/eid1601.090852 PMC2874364

[B14] Zarrilli R Pournaras S Giannouli M Tsakris A Global evolution of multidrug-resistant *Acinetobacter baumannii* clonal lineages Int J Antimicrob Agents 2013 41 11 19 10.1016/j.ijantimicag.2012.09.008 23127486

[B15] Mussi MA Limansky AS Viale AM Acquisition of resistance to carbapenems in multidrug-resistant clinical strains of *Acinetobacter baumannii*: natural insertional inactivation of a gene encoding a member of a novel family of beta-barrel outer membrane proteins Antimicrob Agents Chemother 2005 49 1432 1440 10.1128/AAC.49.4.1432-1440.2005 15793123 PMC1068641

[B16] Vashist J Tiwari V Kapil A Rajeswari MR Quantitative profiling and identification of outer membrane proteins of beta-lactam resistant strain of *Acinetobacter baumannii* J Proteome Res 2010 9 1121 1128 10.1021/pr9011188 20041708

[B17] CLSI Performance standards for antimicrobial susceptibility testing; twenty-first informational supplement 2011 Wayne, PA: M100-S21. Clinical and Laboratory Standards Institute

[B18] Lee K Kim CK Yong D Jeong SH Yum JH Seo YH Docquier JD Chong Y Improved performance of the modified hodge test with MacConkey agar for screening carbapenemase-producing gram-negative bacilli J Microbiol Methods 2010 83 149 152 10.1016/j.mimet.2010.08.010 20801167

[B19] Tenover FC Arbeit RD Goering RV Mickelsen PA Murray BE Persing DH Swaminathan B Interpreting chromosomal DNA restriction patterns produced by pulsed-field gel electrophoresis: criteria for bacterial strain typing J Clin Microbiol 1995 33 2233 2239 7494007 10.1128/jcm.33.9.2233-2239.1995 PMC228385

